# Effect of anti-biofilm peptide *CRAMP-34* on the biofilms of *Acinetobacter lwoffii* derived from dairy cows

**DOI:** 10.3389/fcimb.2024.1406429

**Published:** 2024-08-15

**Authors:** Lin Liu, Hui Li, Chengjun Ma, Jingjing Liu, Yang Zhang, Dengfeng Xu, Jing Xiong, Yuzhang He, Hongzao Yang, Hongwei Chen

**Affiliations:** ^1^ College of Veterinary Medicine, Southwest University, Chongqing, China; ^2^ National Center of Technology Innovation for Pigs, Chongqing, China; ^3^ Immunology Research Center, Medical Research Institute, Southwest University, Chongqing, China; ^4^ Chongqing Academy of Animal Sciences, Chongqing, China; ^5^ Traditional Chinese Veterinary Research Institute, Southwest University, Chongqing, China

**Keywords:** dairy mastitis, anti-biofilm peptide, *Acinetobacter lwoffii*, biofilms, transcriptomic

## Abstract

Dairy mastitis is one of the most common diseases in dairy farming, and the formation of pathogenic bacteria biofilms may be an important reason why traditional antibiotic therapy fails to resolve some cases of dairy mastitis. We isolated and identified three strains of *A. lwoffii* were with strong biofilm forming ability from dairy cow mastitis samples from Chongqing dairy farms in China. In order to investigate the effect of novel anti-biofilm peptide CRAMP-34 on *A.lwoffii* biofilms, the anti-biofilm effect was evaluated by crystal violet staining, biofilms viable bacteria counting and confocal laser scanning microscopy (CLSM). In addition, transcriptome sequencing analysis, qRT-PCR and phenotypic verification were used to explore the mechanism of its action. The results showed that CRAMP-34 had a dose-dependent eradicating effect on *A. lwoffii* biofilms. Transcriptome sequencing analysis showed that 36 differentially expressed genes (11 up-regulated and 25 down-regulated) were detected after the intervention with the sub-inhibitory concentration of CRAMP-34. These differentially expressed genes may be related to enzyme synthesis, fimbriae, iron uptake system, capsular polysaccharide and other virulence factors through the functional analysis of differential genes. The results of subsequent bacterial motility and adhesion tests showed that the motility of *A.lwoffii* were enhanced after the intervention of CRAMP-34, but there was no significant change in adhesion. It was speculated that CRAMP-34 may promote the dispersion of biofilm bacteria by enhancing the motility of biofilm bacteria, thereby achieving the effect of eradicating biofilms. Therefore, these results, along with our other previous findings, suggest that CRAMP-34 holds promise as a new biofilm eradicator and deserves further research and development.

## 1 Introduction

Mastitis is a common disease with high incidence in dairy cows, which seriously affects the health and economic value of dairy cows and is generally caused by pathogenic microorganisms. The most-frequently isolated bacteria in milk samples were *Escherichia coli*, *Streptococcus* spp., and *Klebsiella* spp (Krebs et al., 2023). In recent years, the isolation rate of *Acinetobacter lwoffii* was high in some dairy farms in China (Cui et al., 2024), but it did not attract enough attention. However, *A. lwoffii* is an opportunistic pathogen in patients with impaired immune systems, and which strong biofilm forming ability (Regalado et al., 2009; Pour et al., 2011; Xu et al., 2013). The establishment of biofilms depends on the ability of bacteria to attach to bovine mammary epithelial cells, and mastitis bacteria growing on biofilms are more resistant to antimicrobials (Cerioli et al., 2018).The ability of pathogens to form biofilms in the mammary gland would be a potential source of persistent or chronic infections (Vasudevan et al., 2003). In addition, the ability to invade mammary epithelial cells and the survival of bacteria in the cells are also related to the pathogenesis of persistent mastitis (Cerioli et al., 2018).

Although antibiotics have played a role in the treatment of cow mastitis, the problem of antibiotic residues and drug resistance has been troubling clinical veterinarians (Liu et al., 2024). Antimicrobial peptides are known as new antibiotic substitutes and have attracted much attention (Xia et al., 2021), mainly because they have a low potential to induce *de novo* resistance and have favorable penetration and inhibition of biofilms (Li et al., 2023; Masihzadeh et al., 2023; Wongchai et al., 2024). Some antimicrobial peptides show excellent effects in anti-biofilms, which are also known as anti-biofilm peptides (de la Fuente-Nunez and Hancock, 2015; Ghoreishi et al., 2022). Our previous studies have shown that the murine antimicrobial peptide CRAMP-34 inhibits biofilm formation and eradicates the preformed biofilms of the biofilms model strain *Pseudomonas aeruginosa* PAO1 (Zhang et al., 2022; Wang et al., 2023). Our extensive experiments have confirmed that CRAMP-34 is an excellent anti-biofilm peptide, and it can also delay the development of other antibiotic (colistin and ciprofloxacin) resistance (Wang et al., 2023). However, whether CRAMP-34 has the same inhibitory effect on *A. lwoffii* biofilms is yet to be clarified.

In the current study, we observed the effect of CRAMP-34 on *A. lwoffii* biofilms with strong biofilm-forming ability isolated from milk samples by crystal violet (CV) staining, biofilm viable bacteria counting and confocal laser scanning microscopy (CLSM). In addition, transcriptome sequencing analysis, qRT-PCR and phenotypic verification were used to explore the mechanism of its action. This work has significant implications for developing new antibiotic alternatives, reducing bacterial resistance and maintaining animal health and food safety.

## 2 Materials and methods

### 2.1 Strains and growth conditions

Three strains of *A. lwoffii were* isolated from clinical samples of dairy cow mastitis, and mastitis milk samples were from three dairy farms including Liangjiang Dairy Farm of Chongqing Tianyou Zongheng Animal Husbandry Development Co., Ltd. Briefly, the bacteria from the cow mastitis samples were first isolated using a dilution coating, then, the isolated single colony bacteria were purified using the conventional repetitive streaking technique (Chen et al., 2023). Finally, the purified single colony bacteria were used the universal primers 27F (5 ‘ -AGAGTTTGATCMTGGCTCAG-3 ′) and 1492R (5 ′ -TACGGYTACCTTGTTACGACTT-3 ′) as primers for 16 S rDNA amplification (GenBank: PP783574.1), the amplified products were tested by agarose gel electrophoresis and sent to Beijing Qingke Biotechnology Co., Ltd. for strain identification (Zhang et al., 2024). The identified *A. lwoffii* was mixed evenly in 60% glycerol broth and stored in -80 °C. *A. lwoffii* were removed from -80°C, incubated overnight at 37°C on Luria-Bertani (LB) agar plates, individual colonies were picked and incubated at 37°C in LB nutrient broth to log growth phase, and resuspended to 1×10^5^- 10^6^ Colony-Forming Units (CFU)/mL as the test bacterial solution (Koti et al., 2024).

### 2.2 The antimicrobial resistance analysis of *A. lwoffii*

Seven common antimicrobials (ampicillin, cefquinime, tetracycline, amikacin, ciprofloxacin, kanamycin, and gentamicin) were selected for the antimicrobial resistance analysis of *A. lwoffii*, which were commonly used in the prevention and treatment of mastitis in dairy cows and effective against gram-negative bacteria. The minimum inhibitory concentration (MIC) value of different classes of antimicrobials was determined using the microbroth dilution method recommended by the Clinical and Laboratory Standards Institute 2017 (CLSI 2017). Briefly, the bacterial solution was incubated overnight until the logarithmic growth phase was reached, and the bacterial solution concentration was measured at a UV spectrophotometer at a wavelength of 600 nm at 1×10^5^-10^6^ CFU/mL, the drug serially diluted with LB broth was then added to the 96-well plate, and finally add the resuspended bacterial solution, while making a negative control of the broth and the drug, and incubate in a 37°C incubator for 16-18 h. *In vitro A. lwoffii* biofilms were performed in 96-well plates, 100 μL of resuspended bacterial solution was added to each well, and the lid was closed and incubated in a 37°C incubator for 24 h. Then each well was washed twice gently with PBS (pH=7.4), and added the medicine diluted in advance with LB broth (dilution range was 256 MIC-1/4 MIC) and incubated at 37°C for 3 h. The optimal action time of CRAMP-34 to clear the biofilms screened in the previous experiment, which is consistent with it here for easy control. The biomass of biofilms were assayed, as described previously with minor modifications (Ma et al., 2024). The supernatant was discarded, sterile phosphate-buffered saline (PBS) was added to wash 3 times, 100 μL of 99% methanol was added to each well for 10 min to fix, followed by air drying, 0.04% CV staining was added to each well for 20 min, then the CV was gently washed 3 times with sterile PBS after aspiration, and then 100 was added μL of 33% acetic acid solution was dissolved for 30 min, absorbance values were measured at optical density 590 nm (OD_590_ nm), and the experiment was replicated 3 times independently.

### 2.3 Analysis of CRAMP-34’s ability to eradicate biofilms

CRAMP-34 (GLLRKGGEKIGEKLKKIGQKIKNFFQKLVPQPEQ) was tested for the effect of eradicating on biofilms using the positive peptide LL-37 and ciprofloxacin lactate as a control (CRAMP-34 and LL-37 were purchased from synthesized by ChinaPeptides Co., Ltd., Shanghai, China, and other antibacterial drugs were purchased from Shanghai Maclean’s Biochemical Technology Co., Ltd., Shanghai Yuanye Biotechnology Co., Ltd., and Meilun Biotechnology Co., Ltd., respectively). The above prepared bacterial solution was added to a 96-well cell culture plate and incubated in a 37°C incubator for 24 h pre-biofilms. Afterwards, the plates were washed three times with PBS. Next, 100 µL of each concentration of CRAMP-34, LL-37, and ciprofloxacin diluted with broth were added to the plate and incubated at 37°C for 3 h (8 MIC-1/2 MIC, CRAMP-34: 31.2 μg/mL, 15.60 μg/mL, 7.80 μg/mL, 3.90 μg/mL, 1.95μg/mL, LL-37: 15.60 μg/mL, 7.80 μg/mL, 3.90 μg/mL, 1.95 μg/mL, 0.975 μg/mL. Ciprofloxacin: 2.00 μg/mL, 1.00 μg/mL, 0.50 μg/mL, 0.25 μg/mL, 0.125 μg/mL). The biomass of biofilms was determined by CV staining method, and trypticase soy agar plates (TSA) were used to count viable bacteria as previously described with minor modifications (Zhang et al., 2022; Wang et al., 2023). Briefly, CV (0.04%) was added in biofilms samples for 20 min, and washing with sterile PBS. Then, 33% acetic acid was used to dissolve the bound CV, and absorbance was measured at OD_590_ nm. Meanwhile, bacteria count assay was used to analyze the number of bacteria in biofilms, the mature biofilms were treated with the above drugs for 3 h, the supernatant was discarded, PBS buffer was washed 3 times, 100 μL of 0.1% Triton-100X was added, pipetting and mixing were mixed to fully dissolve the biofilms, and 20 μL was piped to the pre-added 180 according to the results of the pre-test, select an appropriate concentration gradient to draw 20 μL dropped on the plate, then set the plate upright for a few minutes, wait for the liquid to dry, and then placed it in a 37°C constant temperature incubator overnight to record the number of bacteria, and follow the principle of regular gradient reduction of each gradient (Zhang et al., 2022; Wang et al., 2023).

### 2.4 Confocal laser scanning microscopy to observe the structure of the biofilms

CLSM is used to observe the three-dimensional image (3D) structure of biofilms as described in previous studies with some modifications (Zhang et al., 2022). In this experiment, 100 μL of test bacterial solution (OD_600 =_ 0.1, 1×10^5^- 10^6^ CFU/mL) was added to a detachable microplate plate, incubated at 37°C for 24 h, and the biofilms were treated with CRAMP-34 at 37°C for 3 h. The biofilms were rinsed with NaCl (0.9% wt/vol) and stained with the Filmtracer™ LIVE/DEAD™ Biofilm Viability Kit (L10316, Molecular Probes, Thermo Fisher Scientific) in the dark for 20 min. Biofilm samples were rinsed with sterile water and then treated with a CLSM (CLSM-800; Zeiss GmbH, Oberkochen, Germany) with Plan-Apochromat 63x/1.40 oil objective. Signals were recorded with the green channel (SYTO9, excitation wavelength 488 nm) and the red channel (PI, excitation wavelength 561 nm). Multiple images with different Z-stacks are superimposed to form a 3D. Images were acquired using ZEN (black version) software. Each experiment was repeated at least three times. BiofilmQ software was used to analyze the fluorescence intensity, number of biofilms, volume, substrate area, surface area and other parameters, and the removal effect of CRAMP-34 on *A. lwoffii* biofilms were comprehensively evaluated.

### 2.5 RNA-seq and qRT-PCR validation

As mentioned above, the three test groups were set up in six-well cell culture plates, the first group was preformed biofilms grown in LB broth, the second group was *A. lwoffii* grown in LB broth (planktonic bacteria), the third group was the biofilms of the preformed biofilms formed after 24 h of LB broth culture treated with 3 h treatment of CRAMP at 1/2 MIC (3.90 μg/mL) concentration. The biofilm samples were washed three times with the same volume of NaCl solution, and added the pre-cooled NaCl solution, scraping with a cell scraper, the planktonic bacteria and cell scraper scraping samples were snap frozen and stored in liquid nitrogen, collected samples from three independent replicates of each group, entrusted Beijing Ovisen Gene Technology Co., Ltd. to complete high-throughput sequencing. Expression levels of genes were analyzed using DESeq software. The threshold for gene expression was expressed as FPKM values (0.1 or 1), and only genes with FPKM> 1 were analyzed in this study. The Kyoto Encyclopedia of Genes and Genomes (KEGG) pathways and Gene Ontology (GO) analysis were performed for the different genes. 7 representative differential genes related to biofilm were selected for qRT-PCR verification using 16S rRNA as the internal reference gene. Primers for qRT-PCR were designed by Invitrogen Inc. (Carlsbad, CA, USA) and listed in **Supplementary Materials** (**Supplementary Table S1**). The RNA extraction and cDNA synthesis were performed using the method of Roudashti and Zhou et al. with some modifications (Roudashti et al., 2017; Zhou et al., 2018). Experiments were repeated at least three times. The relative expression levels of each target gene were detected by 2 ^−ΔΔCt^ method.

### 2.6 Effect of CRAMP-34 on motility and adhesion of *A. lwoffii*

Transcriptomic analysis found that the expression of some genes related to bacterial pili was up-regulated or down-regulated after the action of CRAMP-34, and the up-regulation or down-regulation of some other genes related to enzymes and proteins also indirectly affected the changes in bacterial motility. Therefore, the effect of CRAMP-34 on the motility and adhesion of bacteria was next verified.

The method of counting bacteria in the upper layer of the biofilms was as described earlier (Zhang et al., 2022; Wang et al., 2023). Here, we increase the bacterial count of the upper layer of other concentrations of the drug that has been carried out to the control for bacterial number changes.

*A.lwoffii* is a type of flagellate-free bacteria whose movement is mainly achieved through the movement of fimbriae, so we verified its twitch movement on a motile agar plate. In the previous experiment, we explored that the most suitable agar concentration for *A. lwoffii* movement was with an agar concentration of 0.1% LB. As mentioned above, we collected the biofilm bacteria, and took 10 μL of vertical and gentle drops on the surface of the agar, and gently moved to a 37°C constant temperature incubator for 48 h when the bacterial solution was slightly volatile and dry and did not flow on the surface (Long et al., 2024).

We took the above-mentioned prepared and enriched biofilm bacteria, diluted them 100 times, and put 100 μL into a new sterile 96-well plate for the control group and the CRAMP-34 intervention group, respectively. Three biological replicates were set for each group. Then, the samples were covered with plastic wrap and cultured in a constant temperature incubator at 37 °C for 3-4 h. After the incubation, the waste liquid was removed, and samples were gently washed twice with PBS, then biofilms were dissolved with 0.1% Triton. Then the number of adherent bacteria was measured by plate colony counting method (Long et al., 2024).

### 2.7 Statistical analyses

Data were analyzed by GraphPad Prism 8.0 software. Student’s t-tests were used to calculate the statistical significance. *P* < 0.05 was considered statistically significant.

## 3 Results

### 3.1 Isolation and identification of bacteria from bovine mastitis samples

All samples were isolated and purified to obtain 32 main pathogens, and the morphological characteristics of the pathogenic bacteria were observed (**Supplementary Figure 1**), and then 16S rDNA amplification was carried out and sent to Beijing Qingke Biotechnology Co., Ltd. for strain identification, and the results showed that there were 13 species of pathogenic bacteria, mainly *Staphylococcus aureus*, *Streptococcus*, *E. coli*, *P. aeruginosa*, *A. lwoffii*, etc. (**Table 1**).

**Table 1:** Species and proportion of main pathogens detected in bovine mastitis samples.

Species of pathogenic bacteria	Number of detected strains	Detection rate (%)
*Staphylococcus aureus*	5	15.63
*Other staphylococci*	2	6.25
*Streptococcus*	1	3.13
*Escherichia coli*	5	15.63
*Klebsiella pneumoniae*	4	12.5
*Pseudomonas aeruginosa*	3	9.38
*Acinetobacter lwoffii*	3	9.38
*Bacillus cloacillus*	3	9.38
*Other bacteria*	6	18.75

### 3.2 Antibiotic sensitivity analysis of *A. lwoffii*

**Figure 1:**
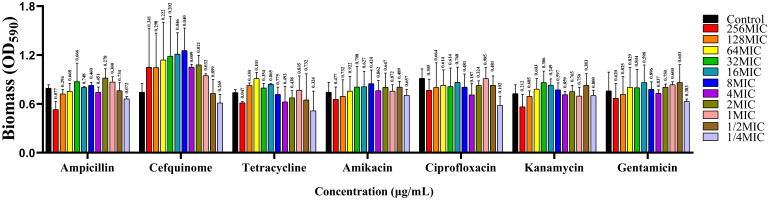
Analysis of antimicrobial resistance of *A. lwoffii* biofilm bacteria. 0, 1/4 MIC, 1/2 MIC, 1 MIC, 2 MIC, 4 MIC, 8 MIC (MIC, minimal inhibitory concentration), respectively, Ampicillin, cefquinime, tetracycline, amikacin, ciprofloxacin, kanamycin, and gentamicin at 16 MIC, 32 MIC, 64 MIC, 128 MIC, and 256 MIC were used to measure OD_600nm_ in *A. lwoffii* biofilms, and OD_600nm_ were used to quantify the biofilms using CV-0.04%. The trial was replicated three times independently. Student’s t-tests were used to calculate the statistical significance. *P* < 0.05 was considered statistically significant.

Antibiotic sensitivity analysis of *A. lwoffii* planktonic cells were showed in **Table 2**. The results showed that *A. lwoffii* planktonic cells were sensitive to most of the selected antibiotics. However, it is worth noting that these antibiotics do not have a significant ability to eradicate the biofilms, even have the ability to promote the formation of the biofilms at certain concentrations (**Figure 1**).

**Table 2:** Antibiotic sensitivity analysis of *A. lwoffii*.

The class of the drug	Drug concentration (μg/mL)	MIC(μg/mL)	Sensitivity
β-lactams	Ampicillin (256)	4-8	S
	Cefquinime (32)	0.063-0.132	S
	Ceftiofur (256)	1-2	S
	Meropenem (2)	0.008	S
Aminoglycosides	Streptomycin (2048)	32	S
	Kanamycin (32)	0.063-0.125	S
	Ampramycin (32)	0.125-0.25	S
	Gentamicin (16)	0.016	S
Tetracyclines	Tetracycline (256)	2	S
Macrolides	Roxithromycin (2048)	8	S
	Azithromycin (256)	0.5-1	S
Amide alcohols	Florfenicol (2048)	16	I
other	Enrofloxacin (256)	1-2	S
	Ciprofloxacin (8)	0.25	S
	Polymyxin (32)	0.25	S
	Amika star (2)	0.031-0.063	S
	Rifampicin (2)	0.031-0.063	S

S, sensitive; I, intermediate; R, resistant.

### 3.3 CRAMP-34 has an eradicating effect on preformed biofilms of *A. lwoffii*

**Figure 2:**
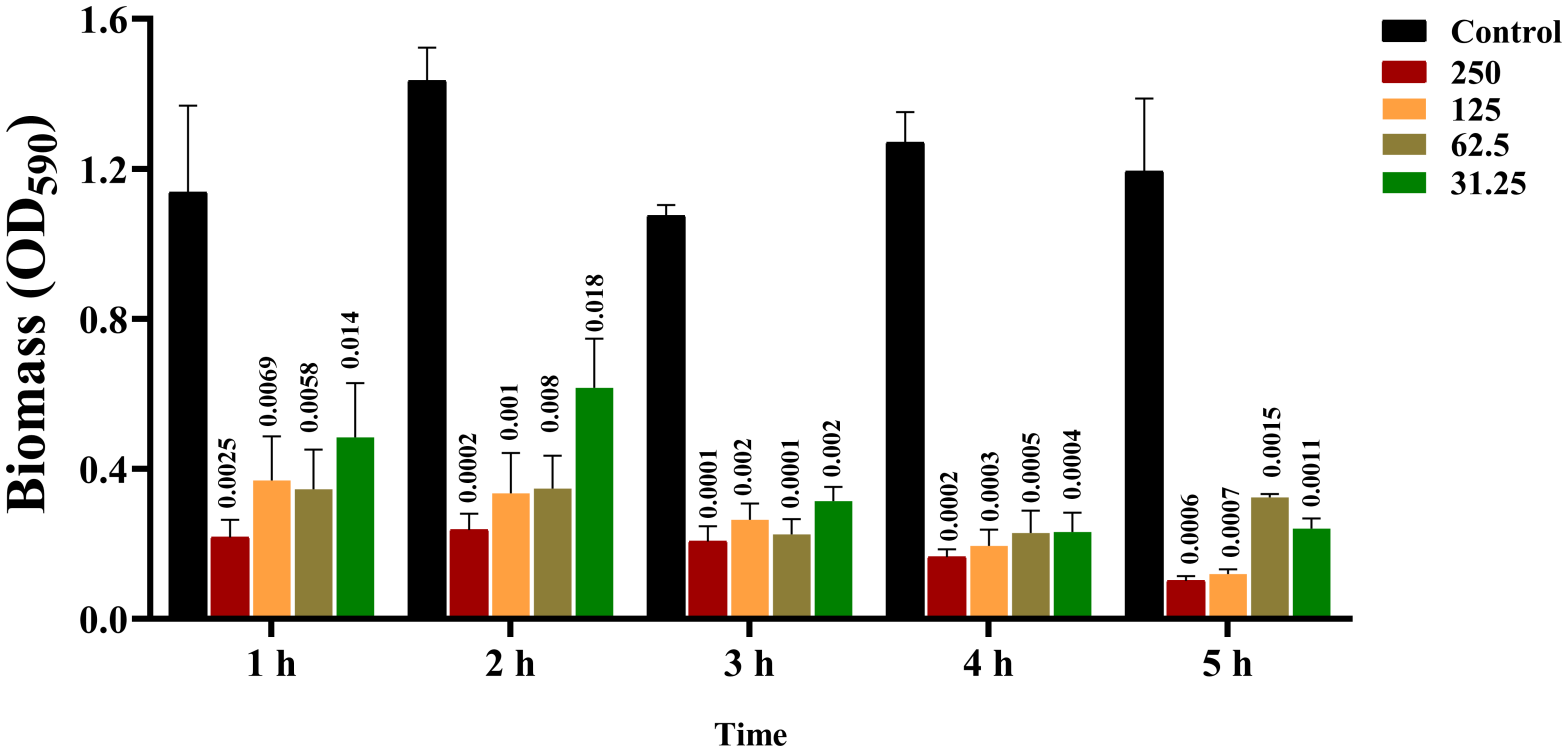
Screening results of CRAMP-34 with optimal action time. 0 μg/mL, 31.25 μg/mL, 62.5 μg/mL, 125 μg/were used at 1 h, 2 h, 3 h, 4 h, and 5 h, respectively. mL and 250 μg/mL of CRAMP-34 were treated with *A. lwoffii* biofilm and quantified using 0.04% crystal violet stainin (CV-0.04%). The trial was replicated 3 times independently. Student’s t-tests were used to calculate the statistical significance. *P* < 0.05 was considered statistically significant.

**Figure 3:**
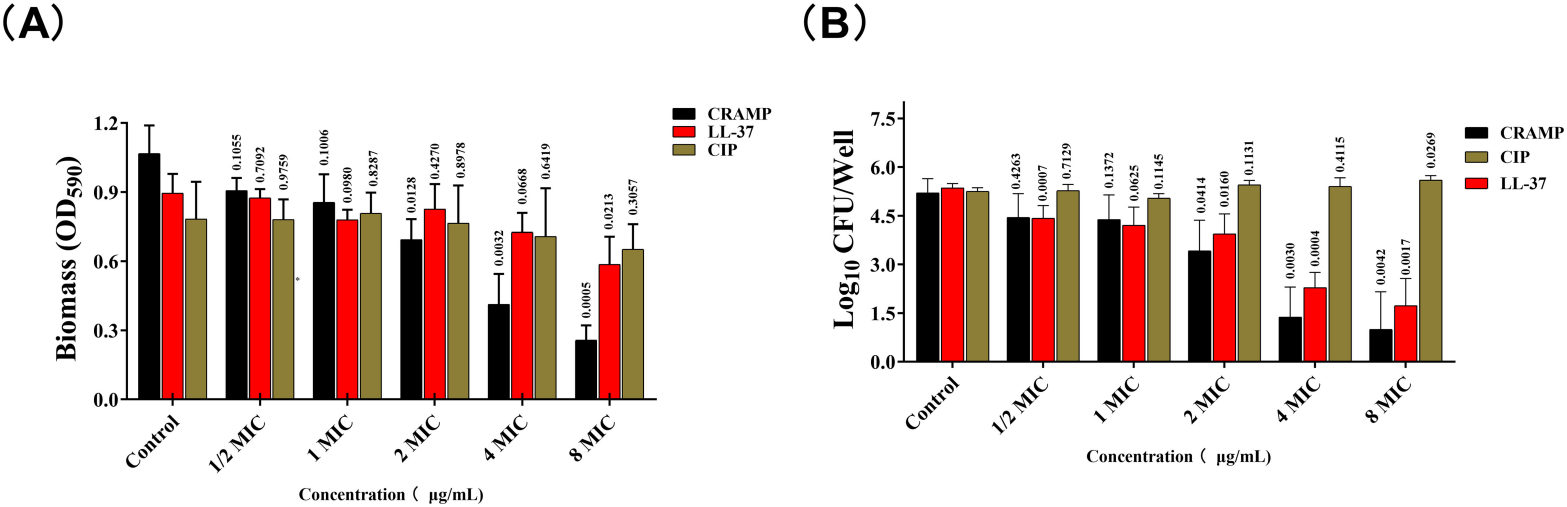
CRAMP-3 inhibits the biofilm activity of *A. lwoffii*. **(A)** Crystal violet staining (CV) was used to determine the biofilm of *A. lwoffii* treated with different concentrations (1/2 MIC-8MIC) of CRAMP-34, ciprofloxacin and LL-37. **(B)** Plate colony count (CC) was used to determine the number of viable biofilms of *A. lwoffii* treated with different concentrations (1/2 MIC-8 MIC) of CRAMP-34, ciprofloxacin, and LL-37. The test was repeated 3 times independently. Student’s t-tests were used to calculate the statistical significance. *P* < 0.05 was considered statistically significant.

The results showed that the effect of CRAMP-34 on the preformed biofilms of *A. lwoffii* was time- and concentration-dependent, with the higher the concentration, the more significant the eradication effect (**Figure 2**). After 3 h of intervention with CRAMP-34, the inhibition rate reached a very significant state, and there was little difference compared with the intervention of 4 h or 5 h. So, 3 h was adopted as the optimal intervention time in follow-up studies. In addition, compared with the commonly used antibiotics ciprofloxacin and human AMP LL-37. The results showed that CRAMP-34 had a more significant antibiofilm effect on *A. lwoffii* biofilms, including biomass and viable bacteria of biofilms (**Figure 3**).

**Figure 4:**
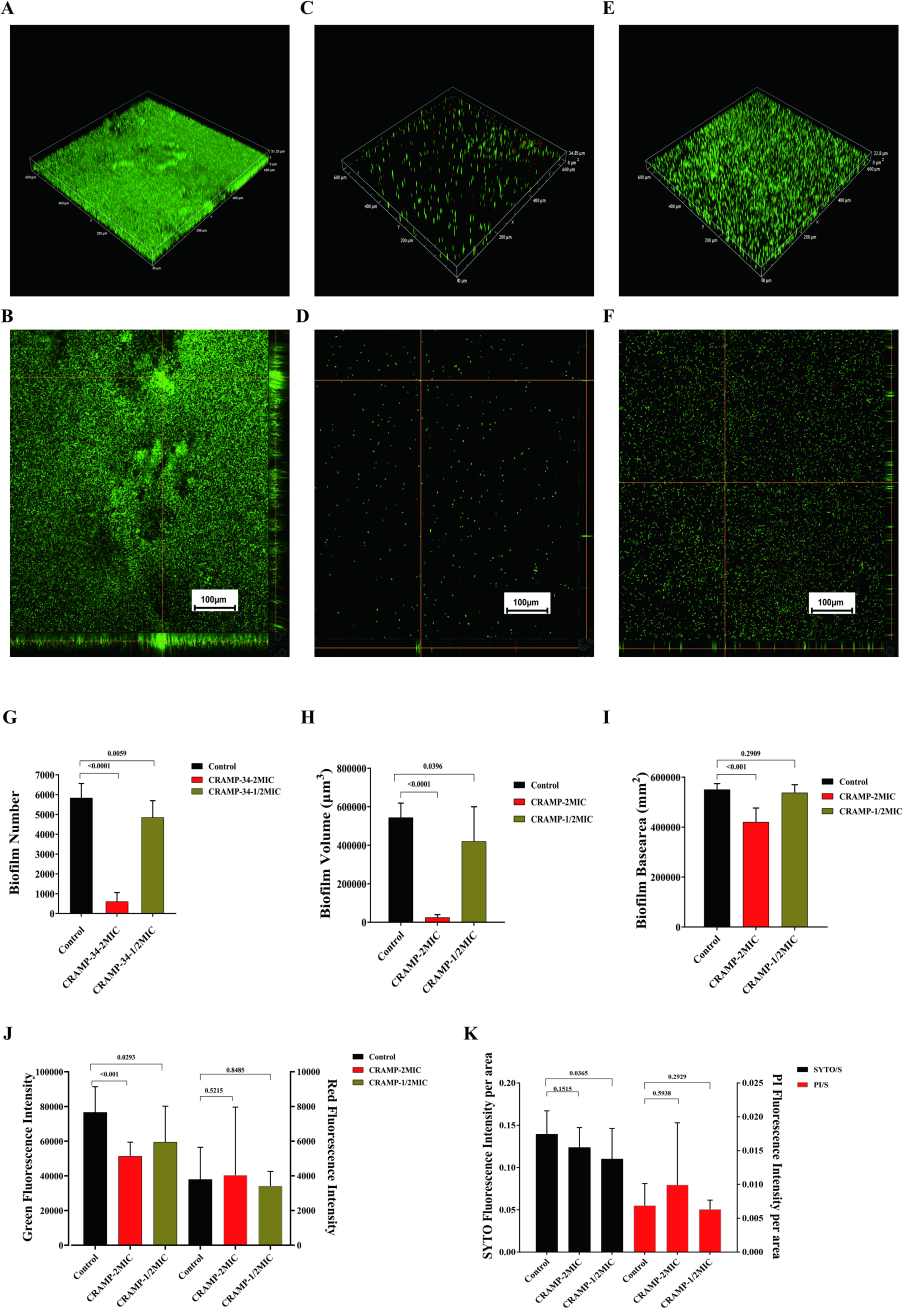
CLSM imaging of *A. lwoffii* before and after CRAMP-34 treatment. **(A, B)** 20× confocal laser scanning microscopy (CLSM) in three-dimensional image (3D) and orthogonal plots of the control group. **(C, D)** 20×CLSM 3D and orthogonal plots of CRAMP-34 treatment group at 2 MIC concentrations. **(E, F)** 3D and orthogonal plots of CRAMP-34 treatment group at 1/2 MIC concentrations of 20× CLSM. **(G)** Number of *A. lwoffii* biofilms treated with CRAMP-34 at 2 MIC and 1/2 MIC concentrations. **(H)** *A. lwoffii* biofilm volume after CRAMP-34 treatment with 2 MIC and 1/2 MIC concentrations. **(I)** Basal area of *A. lwoffii* biofilm after CRAMP-34 treatment with 2 MIC and 1/2 MIC concentrations. **(J)** Fluorescence intensity of *A. lwoffii* biofilm after treatment with cramp-34 at 2 mic and 1/2 mic concentrations. **(K)** Area ratio of *A. lwoffii* bioenvelope after CRAMP-34 treatment with 2 MIC and 1/2 MIC. The images were taken from three random shooting fields in three independent replicate experiments, and the fluorescence intensity, biofilm volume, and biofilm substrate area were analyzed and processed by the open-source software Biofilm Q. Statistical analysis was performed using Prism 8.0 software. One-way ANOVA and LSD multiple test were used to calculate the statistical significance.

To observe the anti-biofilm activity of CRAMP-34, CLSM was applied after live (SYTO 9) and dead (PI) staining of *A. lwoffii* biofilms (**Figures 4A–F**). The results showed that at a concentration of 15.625 μg/mL, CRAMP-34 extremely decreased *A. lwoffii* biofilms with a reduction in the number, volume, and area of biofilms number (*P <*0.001), and the number, volume, and area of *A. lwoffii* biofilms were markedly decreased by CRAMP-34 at the concentration of 7.8125 μg/ml (*P <*0.05). There was no significant difference in red fluorescence between the two groups under the condition of 15.625 μg/mL, the basal surface of the biofilms was positively and significantly decreased (*P <*0.001), and there was no significant difference in the basal area of the biofilms after the intervention of 7.8125 μg/mL (**Figures 4G–K**).

### 3.4 Transcriptome analysis

**Figure 5:**
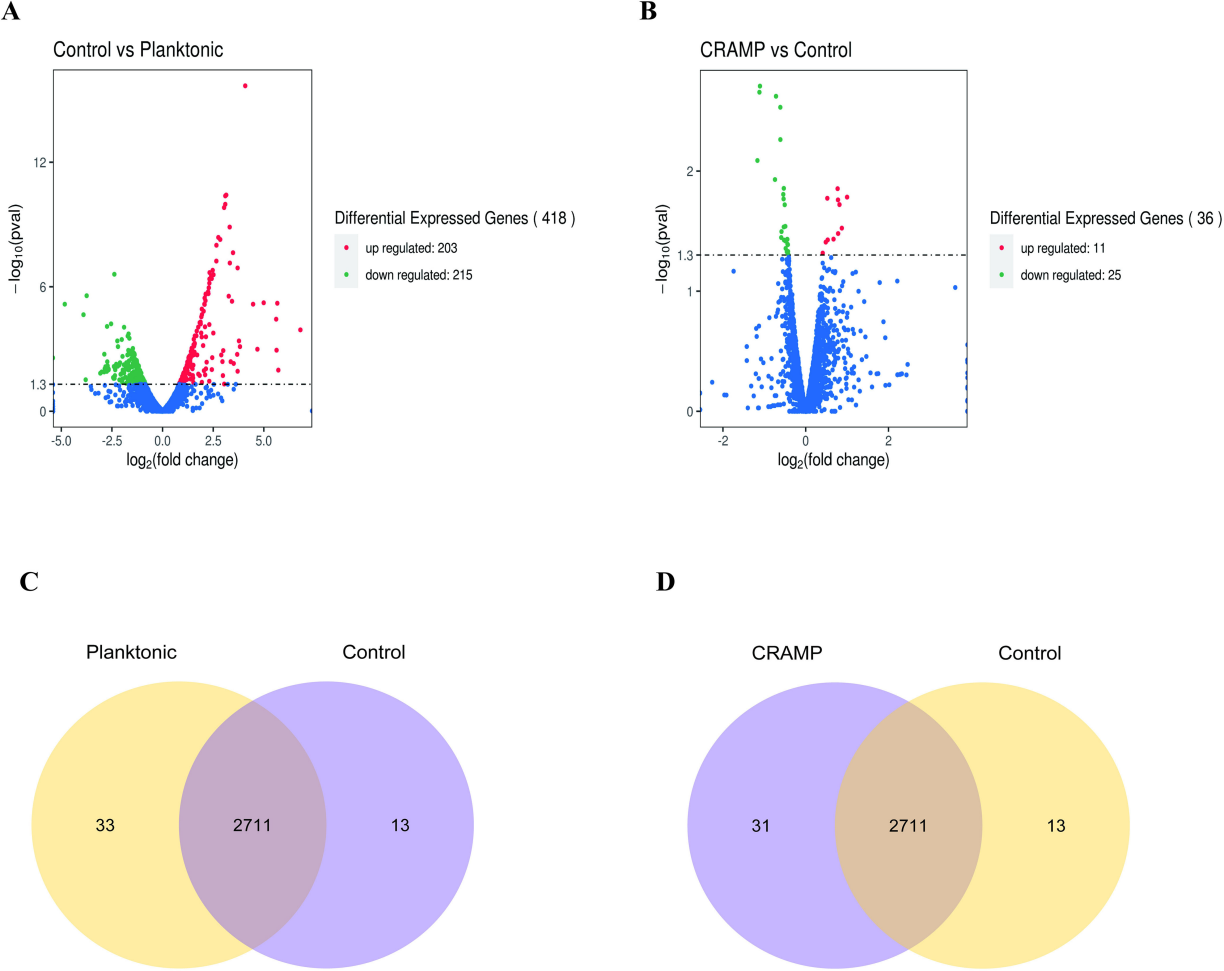
Differential genes in *A. lwoffii* biofilm before and after CRAMP-34 treatment. **(A)** Volcanic diagram of differential genes between *A. lwoffii* biofilm control group and plankton group. **(B)** Differential genes in *A lwoffii* biofilm before and after 1/2 MIC CRAMP-34 treatment. The red dots indicate genes whose expression is upregulated, the green dots indicate genes whose expression is downregulated, and the blue dots indicate genes whose expression has not changed significantly. **(C, D)** Venn diagram of association analysis of differentially expressed genes between groups.

**Figure 6:**
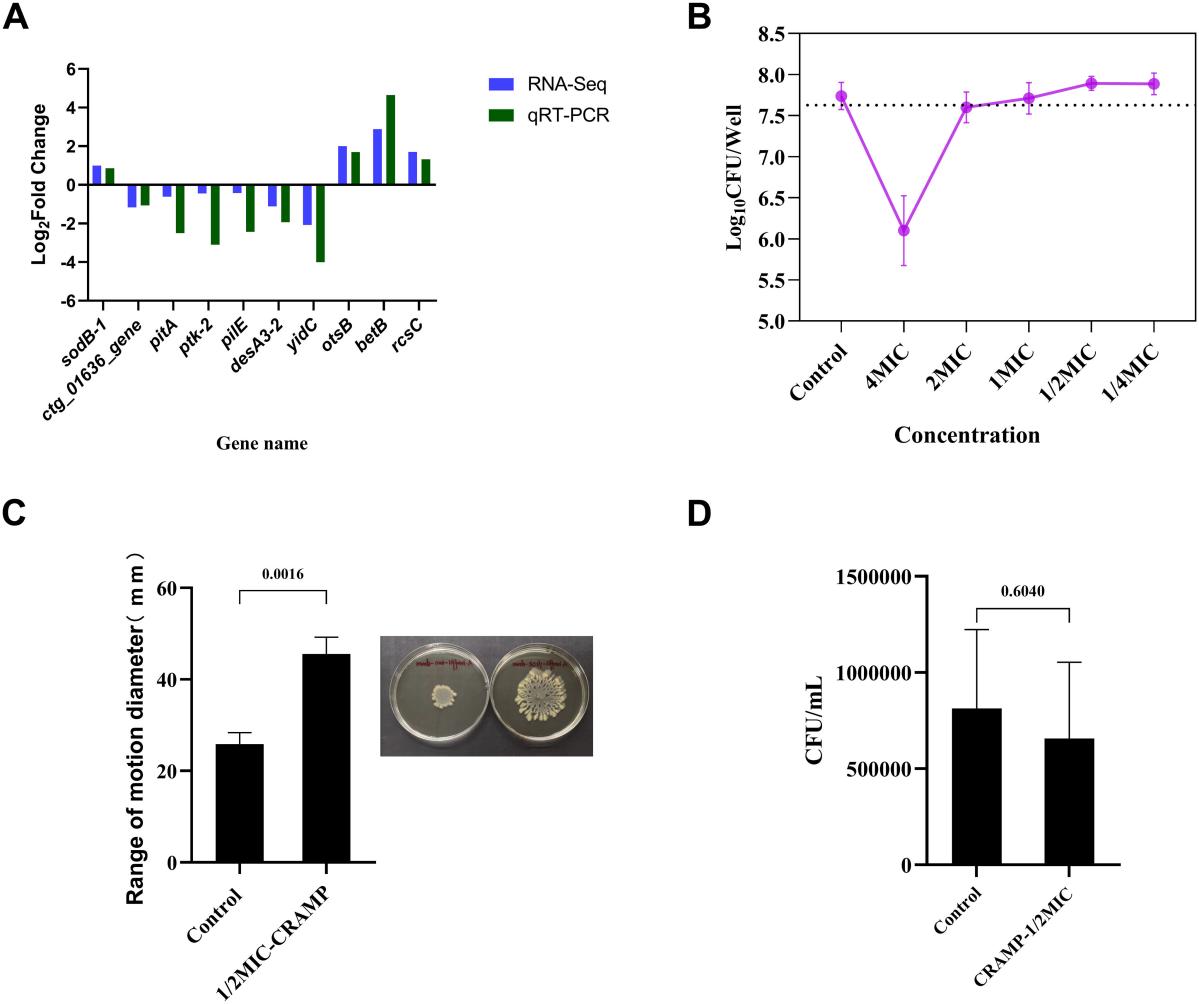
Validation of qRT-PCR and related phenotypic. **(A)** Validation of differentially expressed genes by qRT-PCR to demonstrate the reliability of transcriptome sequencing. Experiments were repeated at least 3 times independently. **(B)** Changes in the number of upper layer bacteria in culture system after the intervention of CRAMP-34 (1/4 MIC-4 MIC) on biofilm. **(C)** The change of movement diameter of biofilm bacteria after treatment with CRAMP-34. **(D)** Number of adhesion of *A lwoffii* on 96-well cell culture plates during the same period of time before and after CRAMP-34 treatment. The trial was replicated three times independently. Student’s t-tests were used to calculate the statistical significance. *P* < 0.05 was considered statistically significant.

The volcano map and Venn diagram were plotted according to the criteria for differential gene screening qvalue<0.05 (**Figure 5**). Compared with the planktonic *A. lwoffii*, there are 84 differentially expressed genes (66 up-regulated and 18 down-regulated) in the *A. loffii* biofilm group, which are mainly involved in the regulation of amino acid biosynthesis and metabolism, biological membrane composition and the composition of ionic transmembrane transporter. The group of CRAMP-34 treated *A. lwoffii* biofilms had 36 differentially expressed genes compared to the untreated group (11 up-regulated and 25 down-regulated), which mainly affected RNA metabolism, polysaccharide biosynthesis, ion binding, transmembrane transporter activity. The histogram of GO enrichment and clustering heatmap of differentially expressed genes was showed in **Supplementary Figures 2** and **Figure 3**. According to the analysis of differential genes, CRAMP-34 may play a role on eradicating the biofilms through the following pathways, including enzyme synthesis, pili, iron uptake system, capsular polysaccharides and other related virulence genes (**Table 3**). Some genes related to biofilm were selected for verification by fluorescence quantitative PCR, and the fluorescence quantification results were consistent with the omics results (**Figure 6A**).

**Table 3:** Biofilm-related genes of differentially expressed.

Gene name	Gene ID	product	Log2FC
Translation of GTPases			
*typA*	ctg_00616_gene	GTP-binding protein TypA/BipA	-0.43194
*lepA*	ctg_00952_gene	Elongation factor 4	-0.42244
*cysN*	ctg_01982_gene	Sulfate adenylyltransferase subunit 1	-0.44659
Pimbima genes			
*pilE*	ctg_00334_gene	Fimbrial protein	-0.42754
Iron uptake system			
–	ctg_01636_gene	NADPH oxidoreductase	-1.1659
*desA*3_2	ctg_01635_gene	NADPH-dependent stearoyl-CoA 9-desaturase	-1.1178
*sodB*_1	ctg_01069_gene	Superoxide dismutase [Mn/Fe]	0.99968
Virulence-related genes			
*ptk*_2	ctg_00080_gene	Tyrosine-protein kinase ptk	-0.44302
*deaD*	ctg_00400_gene	ATP-dependent RNA helicase DeaD	-0.61323
*putA*	ctg_01526_gene	Bifunctional protein PutA	-0.455
*pitA*	ctg_02031_gene	Low-affinity inorganic phosphate transporter 1	-0.61193
other			
*betB*	01421	NAD/NADP-dependent betaine aldehyde dehydrogenase	2.881
*otsB*	02085	Trehalose-6-phosphate phosphatase	2.0008
*yidC*	02799	Membrane protein insertase YidC	-2.0796
*rcsC-3*	02286	Sensor histidine kinase RcsC	1.7063

### 3.5 Validation of motility related phenotypes of *A. lwoffii* by CRAMP-34

The results showed that under the intervention of high concentration of CRAMP-34, the number of live bacteria in the upper layer was smaller than that in the control group. The number of viable bacteria was more than 99% less than that in the control group at the concentration of 4 MIC (1.64 Log_10_ CFU/Well reduction). The number of viable bacteria decreased by 0.14 Log_10_ CFU/Well when the concentration of 2 MIC was applied, and the number of viable bacteria was almost unchanged at the concentration of 1 MIC (decreased by 0.03 Log_10_ CFU/Well). However, when the subinhibitory concentration interfered with the biofilms, the bacteria in the upper layer began to increase, increasing by 0.15 Log_10_ CFU/Well at both 1/2 MIC and 1/4 MIC concentrations (**Figure 6B**).

On the surface of the soft agar, the diameter of the convulsive movement of the bacteria reflects the strength of the bacterial motility after the drug intervention. The results showed that the diameter of bacterial movement after CRAMP-34 treatment was 1.76 times that of control group (*P <*0.01) (**Figure 6C**).

The adhesion of biofilm bacterial was reflected by the number of bacteria adhering to the 96-well plate over the same time period, and the results showed no significant difference in the adhesion of biofilm bacterial after the application of CRAMP-34 compared to the control group (*P* >0.05) (**Figure 6D**).

## 4 Discussion

The formation of biofilms by bacteria has led to the reduction or even ineffectiveness of traditional antibiotics against bacterial infections, which poses a serious challenge to the human and animal health industry (Rather et al., 2021). Bovine mastitis is the most influential disease in the dairy industry, but due to the irrational use of antibiotics in the breeding industry in the past, many dairy cow mastitis pathogens have developed drug resistance, making the recurrence of dairy cow mastitis incurable, and it is urgent to find drugs that can replace traditional antibiotics (El-Sayed and Kamel, 2021). In this study, we isolated and identified 13 pathogenic bacteria from 21 samples of dairy cow mastitis, mainly including *S. aureus*, *E. coli*, *Streptococcus*, *P. aeruginosa*, *A. lwoffii* and *Klebsiella pneumoniae*, indicating that a variety of conditionally pathogenic bacteria are becoming the main pathogens of mastitis in dairy cows. Biofilm bacterial infection leads to the development of various chronic infectious diseases (Zhang et al., 2021), biofilm infection plays a key role in mastitis in dairy cows, and continued treatment with conventional antibiotics is associated with an increased risk of drug resistance, which makes the clinical treatment of bovine mastitis a great challenge (Pedersen et al., 2021).

We identified the film-forming ability of the main pathogenic organisms by CV staining, and screened out 3 strains of *A. lwoffii* with strong biofilm-forming ability. At the same time, it was also found that the biofilm formation ability of *A. lwoffii* was the strongest among all isolates. It is important to note that *A. lwoffii* belongs to the genus *Acinetobacter*, a ubiquitous conditionally pathogenic bacterium that is a normal microflora of the normal skin, oropharynx, and perineum (Ku et al., 2000), which is increasingly reported as a pathogen associated with nosocomial infections such as sepsis, pneumonia, meningitis, urinary tract infections, cutaneous, gastroenteritis, and wound infections (Rakitin et al., 2021). Studies have shown that *A. lwoffii* is a potentially drug-resistant flora, but there are few studies on the resistance mechanism of its bacterial biofilms (Elham and Fawzia, 2019). Subsequently, we selected drugs that are commonly used in clinical practice for the treatment of mastitis and effective against gram-negative bacteria, and carried out antimicrobial resistance analysis against the planktonic bacteria and biofilm bacteria of *A. lwoffii*. The results showed that planktonic *A. lwoffii* were sensitive to most antibiotics, but the higher concentration of some antibiotics not only can not eradicate the biofilms, in some concentrations even promote the formation of biofilms. It has been reported in the literature that antibiotics can stimulate and promote the formation of bacterial biofilms, and sub-inhibitory concentrations of antibiotics may even lead to the formation of thicker bacterial biofilms (Wu et al., 2014). In addition, it was reported that antimicrobial drugs may regulate the physiological state of bacteria by regulating the expression of genes, stimulate bacterial toxicity, and cause serious disease infections (Gao et al., 2022). These highlights the urgent need to develop alternatives to antibiotics to combat infections caused by bacterial biofilms.

At present, there are few studies on the mechanism of drug resistance of *A. lwoffii*, but it has aroused attention in the field of human medicine, and should be paid more attention to its harm in the field of veterinary medicine. In order to combat the harm of bacterial resistance, many researchers are committed to finding alternatives to traditional antibiotics, among which anti-biofilm peptides are an ideal alternative (Luo and Song, 2021). At present, the anti-biofilm peptide LL-37 discovered in human has been widely used in antimicrobial studies (Golla et al., 2020). In our previous research, we reported CRAMP-34 obtained by modifying murine anti-biofilm peptides has a strong ability to clear biofilm (Zhang et al., 2022). In this study, it was showed that CRAMP-34 was better than the positive peptide LL-37 in eradicating the preformed biofilms, while ciprofloxacin and gentamicin had almost no effect on the biofilms, which indicated that traditional antimicrobial drugs did have limitations in eradicating the preformed biofilms. Subsequently, after expanding the culture system in 6-well cell culture plates, it was found that CRAMP-34 still had a significant effect on clearing the biofilms even under the condition of sub-inhibitory concentration, indicating that CRAMP-34 had obvious ability to remove *A. lwoffii* biofilms in a certain concentration range. These results indicated that CRAMP-34 had a strong ability to eradicate *A. lwoffii* biofilms, and the eradication ability was concentration-dependent. The results of CLSM also showed that CRAMP-34 eradicated *A. lwoffii* biofilms mainly by reducing the number and volume of biofilms, but the relevant mechanism was still unclear. Considering the safety and efficacy of CRAMP-34, we chose a lower effective concentration to carry out a follow-up study, i.e., CRAMP-34 at 1/2 MIC.

In order to better explore the mechanism of eradicating biofilms by CRAMP-34, we investigated differentially expressed genes by transcriptome sequencing. By analyzing the transcriptome sequencing results, we found that compared with the control group, there were 84 differentially expressed genes in the planktonic cells group, which were mainly involved in the regulation of amino acid metabolism in biological processes and ion transmembrane transport in molecular functions. There were 36 significantly different genes in the sub-inhibitory concentration of CRAMP-34 intervention group, and the genes related to the biofilm were classified and sorted, and it was found that the sub-inhibitory concentration of CRAMP-34 mainly regulated the gene expression of enzyme synthesis, pili, iron uptake system, virulence genes and other pathways after intervening in the biofilm of *A. lwoffii*. Subsequently, through in-depth analysis of differential genes, we found a number of significantly differential genes that mainly regulate enzyme and protein synthesis, proteins are the bearers of functions in life activities, and their synthesis depends on several translational GTPases (trGTPases), and in this study, three down-regulated genes regulate the synthesis of this class of enzymes, namely the *typA* (BipA) protein regulated by *typA* genes elongation factor 4 regulated by the l*epA* gene and CysN (adenosyl transferase sulfate subunit 1) regulated by the *cysN* gene. According to Goh et al., BipA is an invert protease that is implicated in bacterial motility, cold shock, stress response, biofilm formation, and toxicity (Goh et al., 2021), BipA can regulate the formation of actin bases in host epithelial cells, flagellar-mediated motility and resistance to host defense mechanisms, suggesting that BipA in bacteria is not only a translating factor, but also a key factor in regulating stress adaptation and pathogenicity (DeLivron and Robinson, 2008). Studies have shown that the BipA protein increases virulence and decreases flagellar-mediated bacterial viability (Grant et al., 2003), and in the study of Eunsil Choi et al., BipA-deleted strains had high motility on plates (Choi and Hwang, 2018). Therefore, we hypothesized that the motility of *A. lwoffii* was also altered after the intervention of CRAMP-34, and our subsequent bacterial motility test also confirmed this hypothesis. In addition, BipA can directly or indirectly regulate the transcription of capsular or LPS-related genes (Choi et al., 2020), and among the differentially expressed genes we screened, ptk-2, which is associated with capsular polysaccharide production, was also significantly down-regulated (Hua et al., 2017), with capsular polysaccharides produced by bacteria that can push bacteria to more trophic and oxygen-rich places and kill competitors *ptk*-encoded tyrosine kinase can promote the synthesis of capsular polysaccharides (Hibbing et al., 2010), which produce adhesin and favor bacterial colonization (Zhu et al., 2022). In this study, *ptk*_2 down-regulation may indicate a decrease in capsular polysaccharide production, resulting in a reduction in the extracellular matrix, which is consistent with the results of our CV experiments treated with CRAMP-34. LepA, a ribosome-associated GTPs enzyme found in bacteria, mitochondria, and chloroplasts, is important for the stability and abundance of many membrane-associated proteins, including Mémpoin MspA, which is involved in nutrient absorption and is essential for drug sensitivity (Fishbein et al., 2020). Hanqing Liu et al. reported that LepA is a highly conserved protein that plays a crucial role in bacterial growth and functional protein synthesis under specific conditions, and is structurally similar to EF-G transferase (Liu et al., 2011). Studies have shown that knocking out the lepA gene can improve the survival rate of *E. coli* when treated with antimicrobial drugs (Li et al., 2014), and in our study, the lepA gene was down-regulated in the CRAMP-34 intervention group at subinhibitory concentrations, suggesting that the lepA gene may affect *A. lwoffii* resistance, but the mechanism of its influence needs to be further studied.

*A. lwoffii* is known to be a flagella-deficient bacterium that relies primarily on type IV pili-mediated twitching or surface-associated locomotion for locomotion. Type IV fimli have been reported in a variety of bacteria, which are associated with bacterial motility, bacterial-bacterial interactions, and bacterial attachment on biotic and abiotic surfaces (Denis et al., 2019). In addition, it has been reported in the literature that type IV fimbria can enhance virulence, promote biofilm formation, and promote horizontal gene transfer in different species of bacteria (Chlebek et al., 2021), and *P.aeruginosa* can rely on type IV pili to continuously extend, attach, and retract to pull itself for convulsive movement (Kuhn et al., 2021). In our study, the gene *pilE*, which is associated with type IV pili, was significantly down-regulated, and pilE can indirectly regulate bacterial motility by promoting the assembly of type IV pili (Nguyen et al., 2015), and the PilE protein can promote the adhesion of bacteria to host cells (Chen et al., 2020). The significant down-regulation of the *pilE* gene may be due to the bidirectional regulatory function of the gene, in which the bioenvelope bacteria are subjected to external stress during drug intervention, and in the face of dispersion pressure from the environment, the bacteria inversely regulate and activate the *pilE* gene of their fimbria in order to maintain their own homeostasis. In the study of Hu et al., the expression of pili can regulate iron homeostasis within bacteria (Hu et al., 2022). In this study, multiple iron-related genes were differentially expressed *desA*3_2, *sodB*_1, and *ctg*_01636_gene, *ctg*_01636_gene, because iron is an essential nutrient for the growth of bacterial pathogens, and iron plays a role in regulating microbial metabolism and physiology in proteins (Malmirchegini et al., 2014) (Malmirchegini et al., 2014). The changes in these genes suggest that CRAMP-34 may have affected the biofilm through iron uptake-related pathways.

Finally, the confirmatory test results of bacterial motility and adhesion showed that after the intervention of CRAMP-34 at sub-inhibitory concentration, the motility of *A. lwoffii* biofilm bacteria were significantly enhanced, the number of viable bacteria in the upper layer increased, and the diameter of the motile range increased by 1.76 times. It is speculated that CRAMP-34 may promote the transformation of biofilm bacteria into planktonic bacteria by increasing the motility of bacteria, leaving a central cavity of the biofilm, and eventually leading to the dispersion of the biofilm. However, the results of the biofilm bacterial adhesion test showed that there was no significant change in the bacterial adhesion after the action of CRAMP-34, indicating that CRAMP-34 had no significant effect on the biofilm reformation ability of *A. lwoffii*. We reviewed the literature to analyze that the reason for the enhanced motility of bacteria may be due to the fact that BipA binds to ppGpp, which can directly control pilial biosynthesis and biofilm formation (Zheng et al., 2017). The ppGpp is an important second messenger in bacterial cells, which promotes bacterial adaptability and repair in adverse environments (Dalebroux et al., 2010), and it has a certain functional correlation with c-di-GMP, and low levels of c-di-GMP promote the diffusion of biofilms. In addition, the increase or decrease of the extracellular matrix, especially exopolysaccharides, is also an important reason for the change in motility. In our study, the gene PTK-2 encoding tyrosine kinase was significantly down-regulated, and tyrosine kinase promoted the synthesis of capsular polysaccharides, suggesting that the enhancement of motility of biofilm bacteria is likely to be directly related to capsular polysaccharides. The increased motility of bacteria leads to the transformation of biofilm bacteria into plankton, leaving a hollow biofilm, which leads to the dispersion of bacterial biofilm. However, the specific reasons why CRAMP-34 increases the movement of biofilm bacteria remain to be further investigated.

## 5 Conclusion

CRAMP-34 has the ability to eradicate *A. lwoffii* biofilms in a concentration-dependent manner, and the sub-inhibitory concentration of CRAMP-34 still has a significant eradicating ability, which mainly affects the gene expression of enzyme synthesis, fimbri, iron uptake system, virulence genes and other pathways. It was speculated that CRAMP-34 may promote the dispersion of biofilm bacteria by enhancing the motility of biofilm bacteria, thereby achieving the effect of eradicating biofilms.

## Data Availability

The original contributions presented in the study are included in the article/**Supplementary Material**. Further inquiries can be directed to the corresponding authors.
