# Supplementary material for: Effect of anti-biofilm peptide CRAMP-34 on the biofilms of Acinetobacter lwoffii derived from dairy cows
Source: Front Cell Infect Microbiol. 2024 Aug 15;14:1406429. doi: 10.3389/fcimb.2024.1406429 (PMC11358070; doi:10.3389/fcimb.2024.1406429)
Supplement: Supplementary file 1 [file DataSheet1.docx]

Supplementary Material

**Table S1 gene qRT-PCR primer sequence**

| **The name of the gene** | **Upstream primers** | **Downstream primers** |
| --- | --- | --- |
| 16S rRNA | GGTGCCTTCGGGAACTTACA | ACCCGCTGGCAAATAAGGAA |
| *pitA* | CCTGTACCGGTGTCAGCTTT | CCGTTTGCGCAGACAATTGA |
| *pilE* | AGCAACAGTGGGTGTATCGG | CCTGTGGTACCTGCTGCTAG |
| *ptk*_2 | GGTGGAAAACGGGTACTCGT | ACTCTTGCCGCGAGTAACAA |
| *desA*3_2 | AATGAGAGCCGTGGTCACTG | CACACCGCCTGAACTTTTGG |
| ctg_01636_gene | GTATGTGGTGGTGAAGGGCA | GAGGCACAGTCATATCGCCA |
| *sodB*_1 | GCAGGAACCGAATGGGAAGA | GGGAGTGATTGGCATGTCCA |
| *yidC* | GTTGACTCAGGGTAATGGTGAAG | CAGTACCTGCAGGCACATTAAAG |
| *otsB* | GGATTGTCTGACCCACTTTCAAG | AATGAGCTTGCTGGTGAATGTG |
| *betB* | ATAAATACCTGGGGCGAATCTCC | GGTCTGGGTATATTGCTGCAAAG |
| *rcsC* | GCGCTATTTTCAGGAACAGACC | TCTGATTGGAACTCAGCGGAAA |


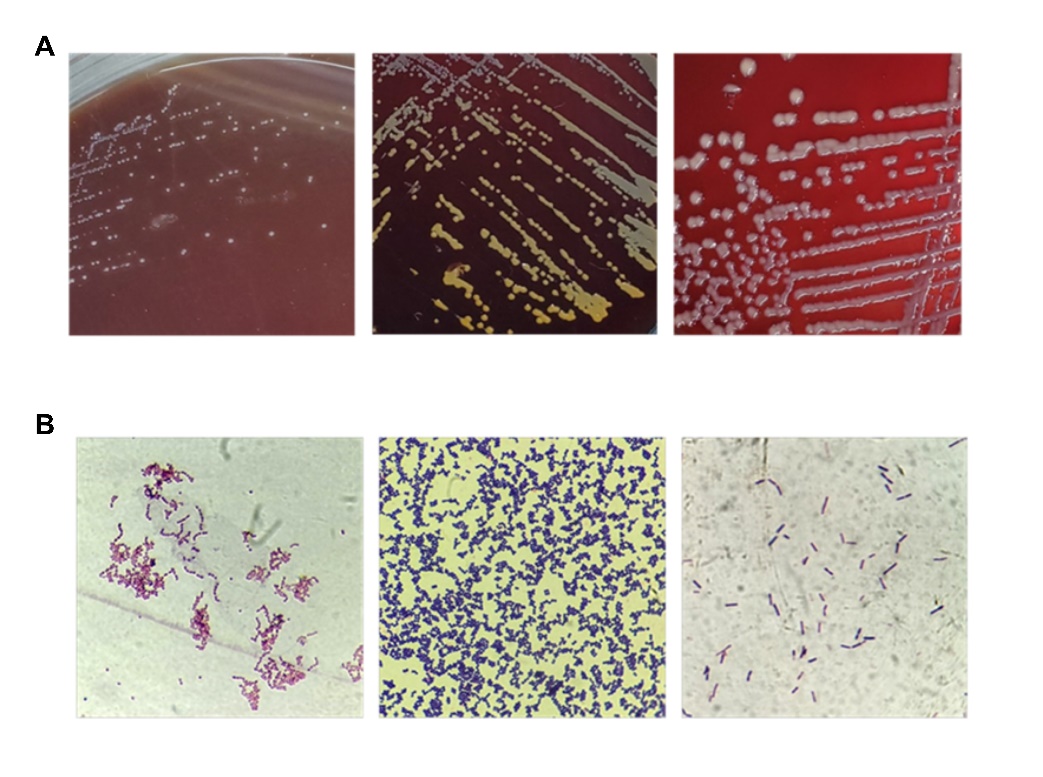


**Supplementary Figure 1**. Samples of mastitis from dairy cows were isolated and identified for morphological results. The samples were isolated from the pathogens, and the main pathogens were purified and microscopically examined, and the morphological characteristics of some isolated bacteria and morphological observations (A) were found to be the characteristics of some isolated bacterial cultures. (B) Morphological observations of some isolates.


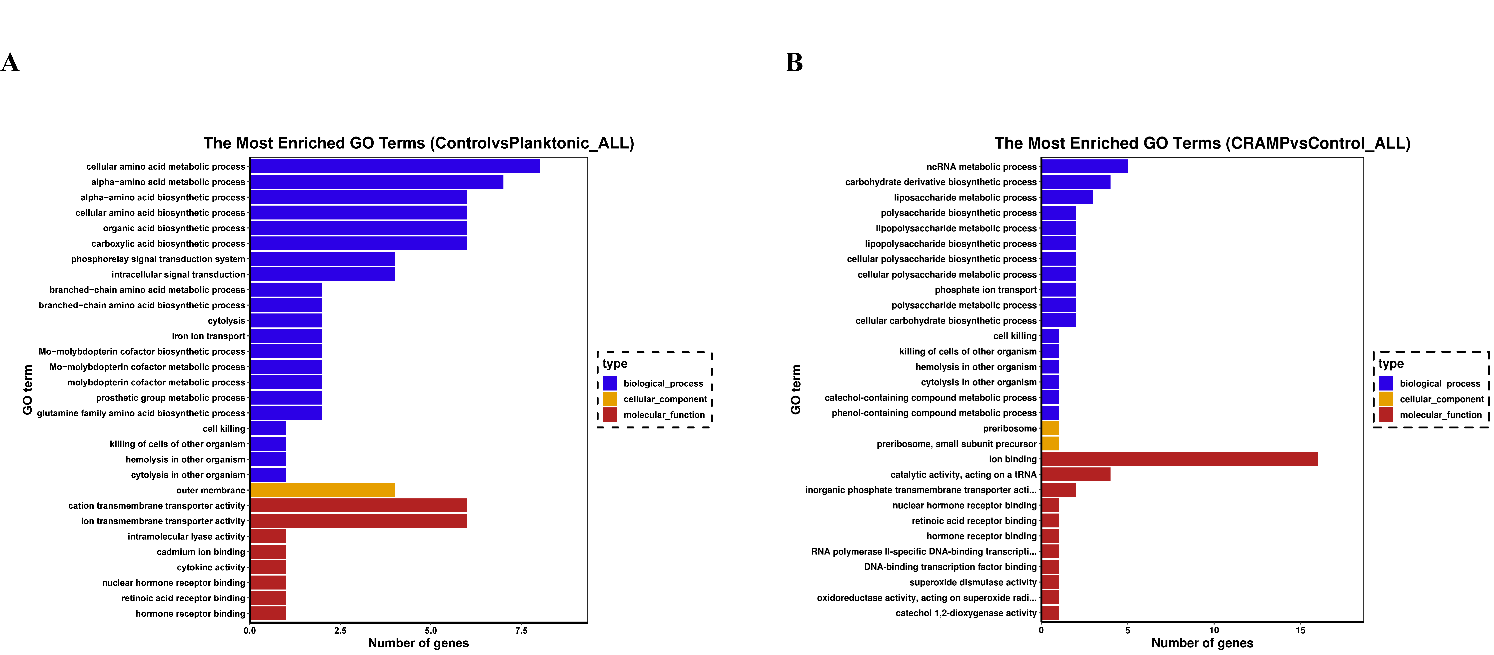


**Supplementary Figure 2**. Histogram of GO enrichment of differentially expressed genes. The experimental group was treated with 3.90 μg/mlcramp-34 for 3 h, and the control group and plankton group were treated with LB broth for 3 h, and a total of 3 completely independent biological sample replicates were collected. (A) Histogram of GO enrichment of differential genes in the biofilm control group and the plankton group. (B) Histogram of GO enrichment of differentially differentiated genes in the biofilm control group and CRAMP-34 treatment group.


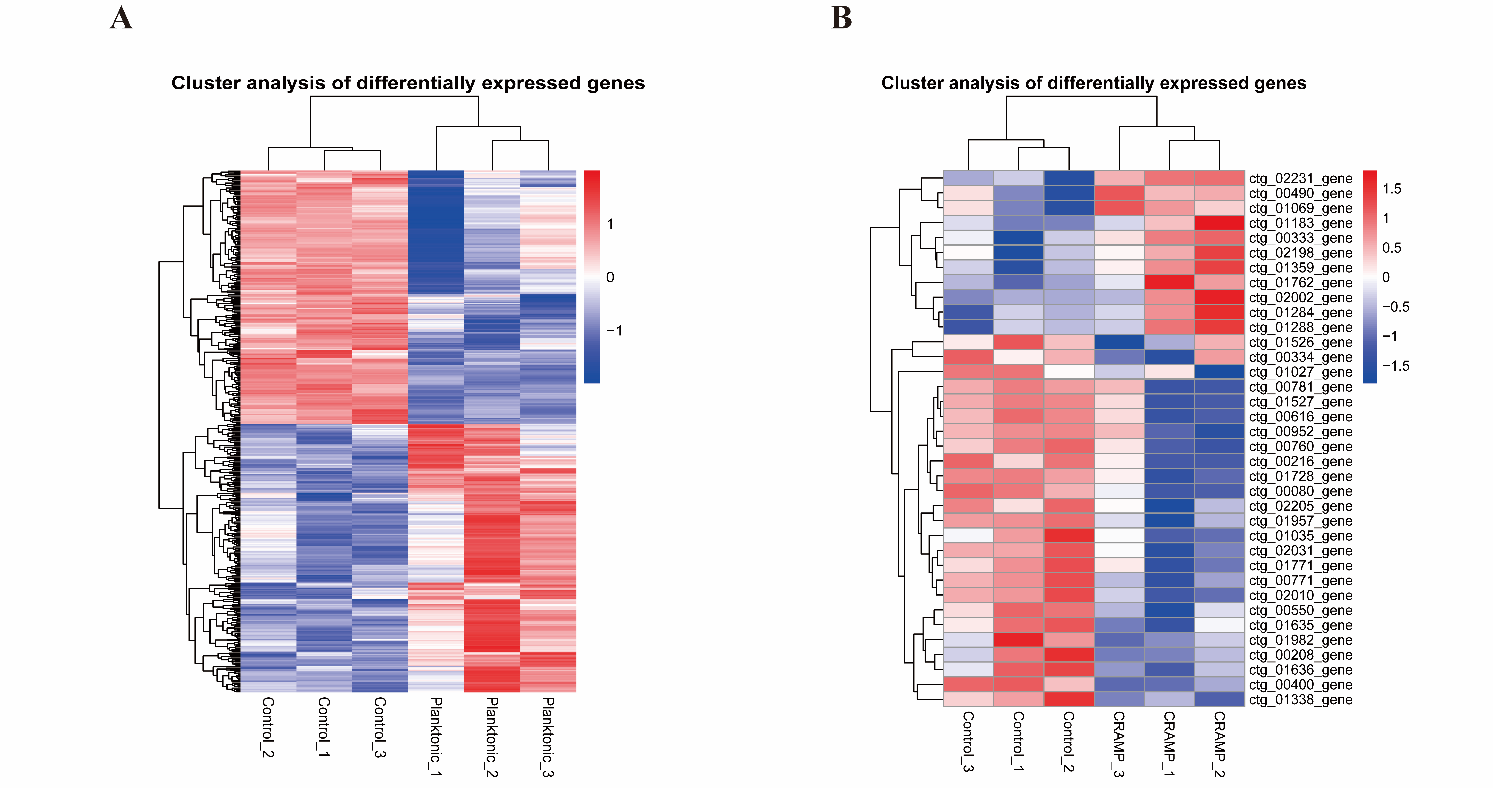


**Supplementary Figure 3.** Transcriptomics differential gene clustering heatmap. The experimental group was treated with 3.90 μg/mlcramp-34 for 3 h, and the control group and plankton group were treated with LB broth for 3 h. (A) Heatmap of differential gene clustering between the biofilm control group and the plankton group. (B) Heatmap of differential gene clustering between the biofilm control group and the CRAMP-34 treatment group.
